# Identifying important ecological areas for potential rainwater harvesting in the semi-arid area of Chifeng, China

**DOI:** 10.1371/journal.pone.0201132

**Published:** 2018-08-22

**Authors:** Hao Zheng, Jixi Gao, Gaodi Xie, Yu Jin, Biao Zhang

**Affiliations:** 1 Institute of Geographic Science and Natural Resource Research, Chinese Academy of Science, Beijing, China; 2 Nanjing Institute of Environmental Sciences, Ministry of Environmental Protection of China, Nanjing, China; 3 College of Apply Meteorology, Nanjing University of Information Science and Technology, Nanjing, China; Old Dominion University, UNITED STATES

## Abstract

Global water shortage is becoming increasingly severe, so the identification and protection of potential areas for harvesting water play important roles in alleviating drought. Suitable sites for potential water harvesting require a high runoff potential. Avoiding soil erosion caused by high surface runoff, however, is also necessary. We therefore developed a procedure for the continuous accounting of runoff potential based on the Soil Conservation Service curve number and potential risks of water and soil loss based on the universal soil loss equation to evaluate the potential for water harvesting. Suitable sites for rainwater harvesting covered 24.90% of the semi-arid area of Chifeng, southeastern Inner Mongolia, China. The best areas accounted for 8.4% of the study area. The southern part of the Greater Hinggnan Mountains in northern Chifeng had a large rainwater harvesting area, and the western and eastern parts of the Chifeng area belonging to Horqin Sandy Land and Hunshandake Sandy Land, respectively, had smaller rainwater-harvesting areas. The eight reservoirs in the Xilamulun River Basin were further analyzed as an example. Derived sites investigated by ground-truth field verification indicated a method accuracy of 87.5%. This methodology could be effective in other areas with similar requirements due to the increasing demand for water resources and requirements for the protection of soil-water resources.

## 1 Introduction

Global climate change and the population explosion have caused severe water shortages in some areas. Liniger and Weingartner (1998) reported that about 90% of water resources were derived from forest catchments in the arid and semi-arid mountainous areas of the world [1]. Researchers are therefore increasingly recommending the collection of rainwater using the vegetation of ecosystems, the establishment of systems for collecting rainwater and an increase in the output of available water resources [2,3]. The Chinese government has continuously strengthened the protection of forest resources in recent years; forest area has increased from 124.6 × 10^6^ ha in 1990 to 208 × 10^6^ ha in 2014. The area of planted forests is the largest in the world. Water crises, however, have become increasingly prominent as the area and density of forests increase, mainly due to the loss of rainwater by canopy interception and evapotranspiration [4–6]. The availability of water is limited during summer due to large fluctuations of precipitation and low capacities to store water, even in areas with high rainfall and low evapotranspiration [7–9]. The per capita water resources in 2016 was 2055 m^3^ in China, one of the 13 countries that suffer most from serious shortages of water resources per capita. Water shortage can be minimized by careful planning and operation. Water stress can be managed by harvesting runoff during rainy days, and this harvested water can be used for social development during dry spells [10]. Identifying potential sites for rainwater harvesting (RWH) is therefore an important step toward maximizing water availability and land productivity in the arid and semi-arid areas of China.

Rainfall, terrain, soil type, land use, runoff characteristics and other indicators have been used to identify potential RWH sites in semi-arid areas [11–14]. Runoff is one of the most important parameters for predicting potential RWH sites [10,14]. The Soil Conservation Service curve number (SCS-CN) method, one of the commonest models for simulating rainfall and runoff due to its simple structure, few parameters and low requirement for empirical data, accounts for many factors, including rainfall, soil properties and land-use type [11]. Many studies have identified potential sites for RWH from estimates of runoff by directly modifying and evaluating the SCS-CN model [10,15–17], and this approach can increase the accuracy of spatial estimates of runoff useful for the precise implementation of RWH structures. High surface runoff, however, can cause soil erosion, i.e. the loss of soil and its nutrients, and can then lead to the formation of sludge layers at the bottom of reservoirs and thus to the deterioration of water quality. The protection of vegetation or engineering measures is thus needed in areas with a high risk of soil erosion to increase infiltration, mitigate surface runoff and increase the capacity of ecosystems to accumulate and store rainwater instead of releasing and acquiring surface runoff. We therefore assessed the risk of soil erosion and used geographical information system software to conduct a superposition analysis of spatial and attribute data for slope, land use, soil type and catchment path to identify important areas for harvesting water resources and to provide an important basis for establishing RWH facilities.

We selected the Chifeng area as a representative semi-arid region of southeastern Inner Mongolia, China, to assess RWH potential. This area has had frequent water shortages due to erratic monsoons, especially with the higher demand for water as the population has increased. The total surface runoff in the early 1980s of several major rivers in the Chifeng area, including Wulijimulun River, Xilamulun River and Laoha River, was nearly 3.3 × 10^9^ m^3^. The total runoff decreased by nearly half after 2000 [18]. The groundwater level decreased by 11.52 m from 2005 to 2014 due to over-exploitation [19]. Average Chifeng per capita water resource is <1000 m^3^, equivalent to 40% that of China [20]. Water shortage has become an important factor restricting local sustainable development, and large amounts of rainwater drain directly into oceans and seas without being used or stored. The total water consumption of Chifeng City is about 2.403 × 10^9^ m^3^, but the total water supply of the reservoirs in the city is only 299 × 10^6^ m^3^. The 41 small and medium-sized reservoirs have nearly been emptied [21]. A better understanding of how to identify RWH areas is therefore crucial for the efficient collection of water resources and for alleviating the water crisis in this area. The objective of this study was thus to identify potential RWH sites in this semi-arid area.

## 2 Materials and methods

### 2.1 Study area

**Fig 1:**
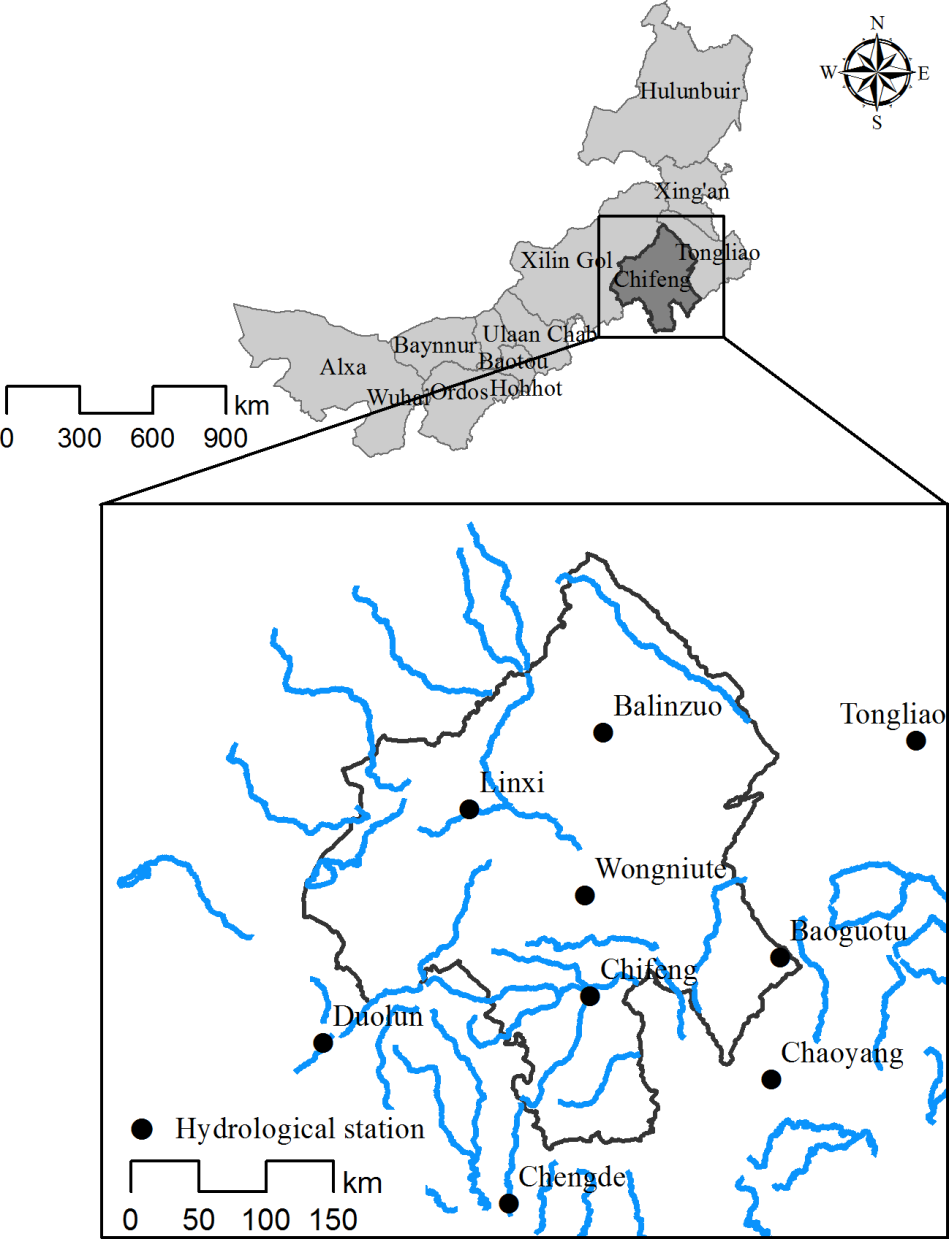
Map of the study area in Inner Mongolia.

The study area (Fig 1) is spread over 90 021 km^2^ in Inner Mongolia, China (41°17′10″-45°24′15″N, 116°21′07″-120°58′52″E). Chifeng is in the southern part of the Greater Higgnan Mountains at the northern foot of Yanshan Mountain. It is surrounded on three sides by mountains, topographically high in the west and low in the east, with geomorphologic features of many mountains and hills at elevations generally between 500 and 1500 m a.s.l. The dominant soil types are montane Phaeozems, Greyzems, Brunisols, and chestnut soils. The study area is in a mid-temperate zone with continental monsoon climatic characteristics. Annual mean temperature is 0–7°C, and the annual amount of sunshine is 2700–3100 h. Rainfall is concentrated in June to August, which accounts for 70–80% of the total annual precipitation. The average annual wetness degree is 0.5–0.8, and the average annual evaporation reaches 1600–2500 mm [20].

### 2.2. Data sources and processing

#### 2.2.1 Meteorological data

We collected data from nine meteorological stations in or near the study area. Rainfall data were monthly averages or for intervals of 30 days based on the assumption of 12 heavy rains per year, and data for surface runoff were similarly organized [22, 23]. Climatic data were derived from the Chifeng Meteorological Bureau [24]. Geographical coordinates for the meteorological stations are shown in Table 1. Rainfall data for the watershed (Chifeng rain-gauge stations) for 1992–2011 were analyzed. The difference in antecedent soil-moisture content was ignored because monthly precipitation data had been collected and the monthly precipitation time of various meteorological stations is synchronous. Antecedent moisture conditions II was considered for the entire watershed for a given storm.

**Table 1:** Geographical coordinates for the nine meteorological stations.

Hydrological station No.	County/city	Latitude (N)	Longitude (E)
1	Balinzuo	43°58'59"	119°24'00"
2	Linxi	43°36'00"	118°04'00"
3	Wongniute	42°55'59"	119°00'00"
4	Chifeng	42°16'10"	118°55'59"
5	Tongliao	43°36'00"	122°16'00"
6	Duolun	4210'59"	116°28'00"
7	Baoguotu	42°19'59"	120°42'00"
8	Chengde	40°58'59"	117°57'00"
9	Chaoyang	41°33'00"	120°27'00"

#### 2.2.2 Slope and Hydrological soil groups

**Fig 2:**
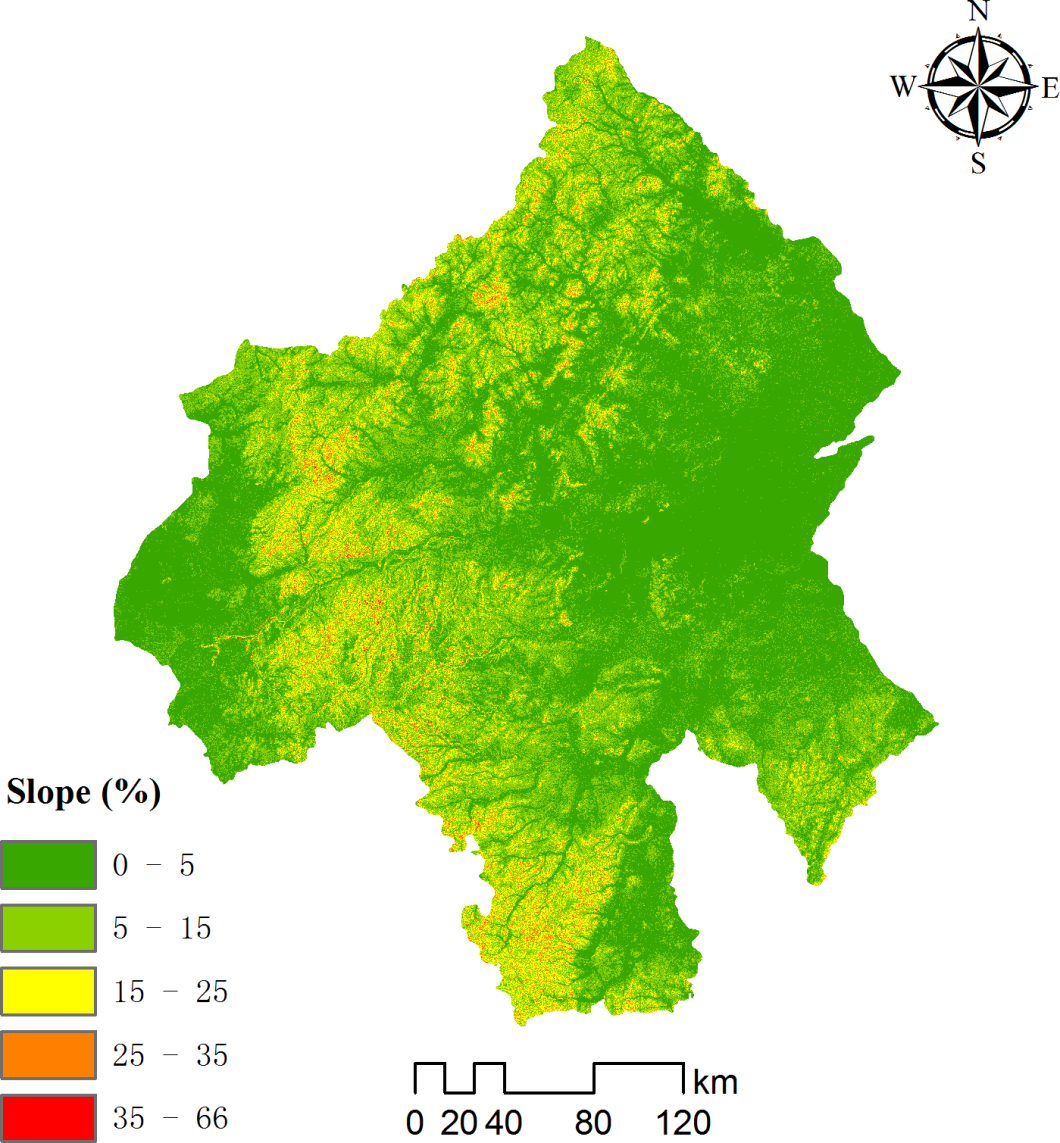
Slope classification of the Chifeng area.

A 30-m pixel digital elevation model (DEM) was used to determine elevation, slope and aspect. The slope data were derived from the ASTRE sensor with a resolution of 30 m. The area of gentle slopes (0–5°) in the Chifeng area accounted for 54.71% of the total study area, the area of slopes of 5–15° accounted for 33.64% and the areas of relatively steep and steep slopes accounted for only 9.94 and 1.59%, respectively (Fig 2). The majority of the study area had slopes <15°, which allowed the retention of water for a longer time, which thus increased infiltration and recharge. These conditions are suitable for harvesting rainwater.

**Fig 3:**
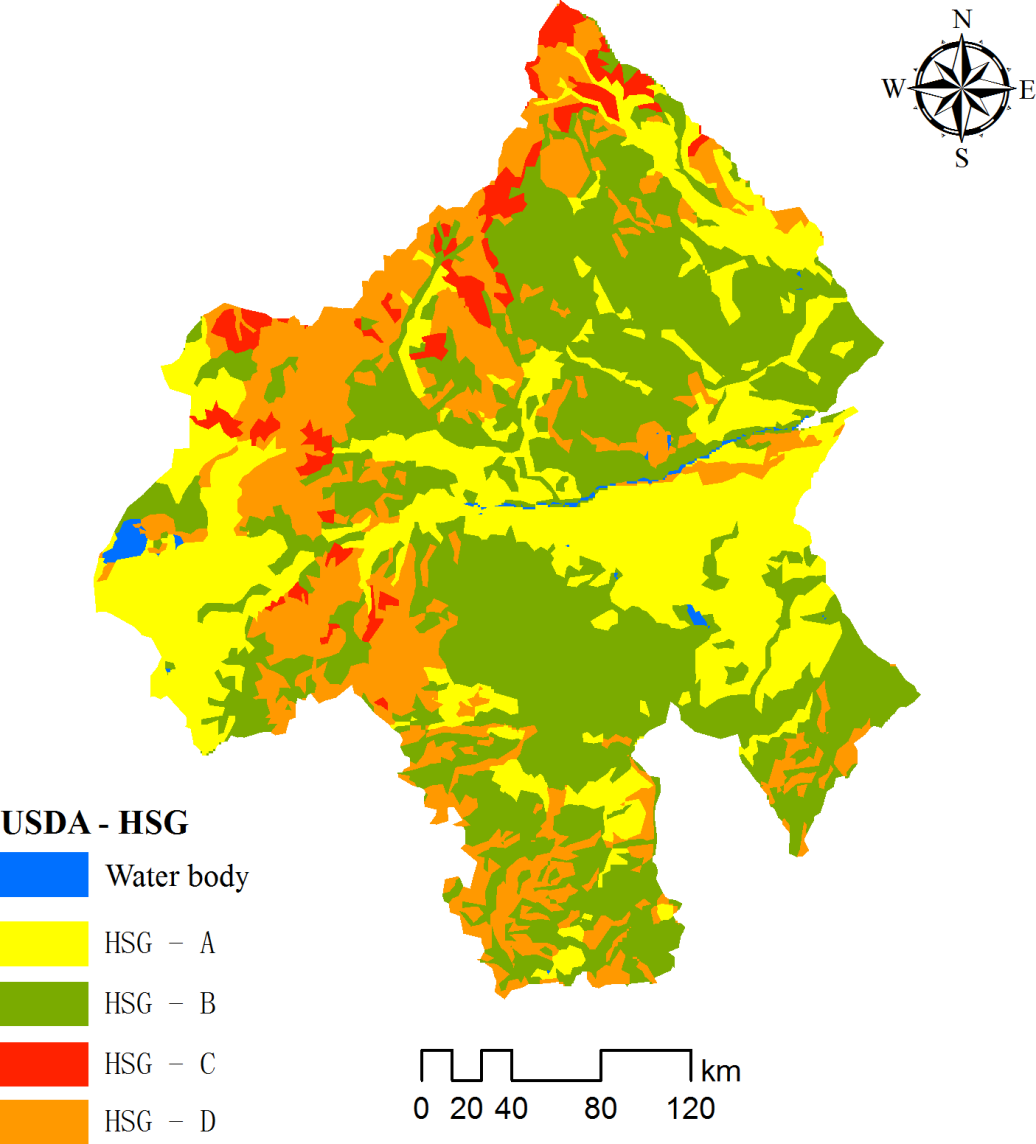
Map of soil types for the study area.

Various soil structures and physicochemical properties can affect surface runoff by influencing infiltration. Data for soil texture were derived from the Harmonized World Soil Database (HWSD) version 1.1, which is compiled by the United Nations Food and Agricultural Organization and the International Institute for Applied Systems Analysis in Vienna, at a scale of 1:1 000 000 [25]. Soil type (stabilized infiltration rate) was reclassified from the United States Department of Agriculture (USDA) [26] Hydrologic Soil Group (HSG) (Fig 3), represented by categories A to D, for processing in the SCS-CN model. HSG-A represents high permeability with low runoff potential, accounting for 27.27% of the study area, HSG-D represents very shallow soil or high clay content with high runoff potential, accounting for 20.66% of the study area and HSG-B and -C are intermediate classes, accounting for 50.59% of the study area.

#### 2.2.3 Land use

**Fig 4:**
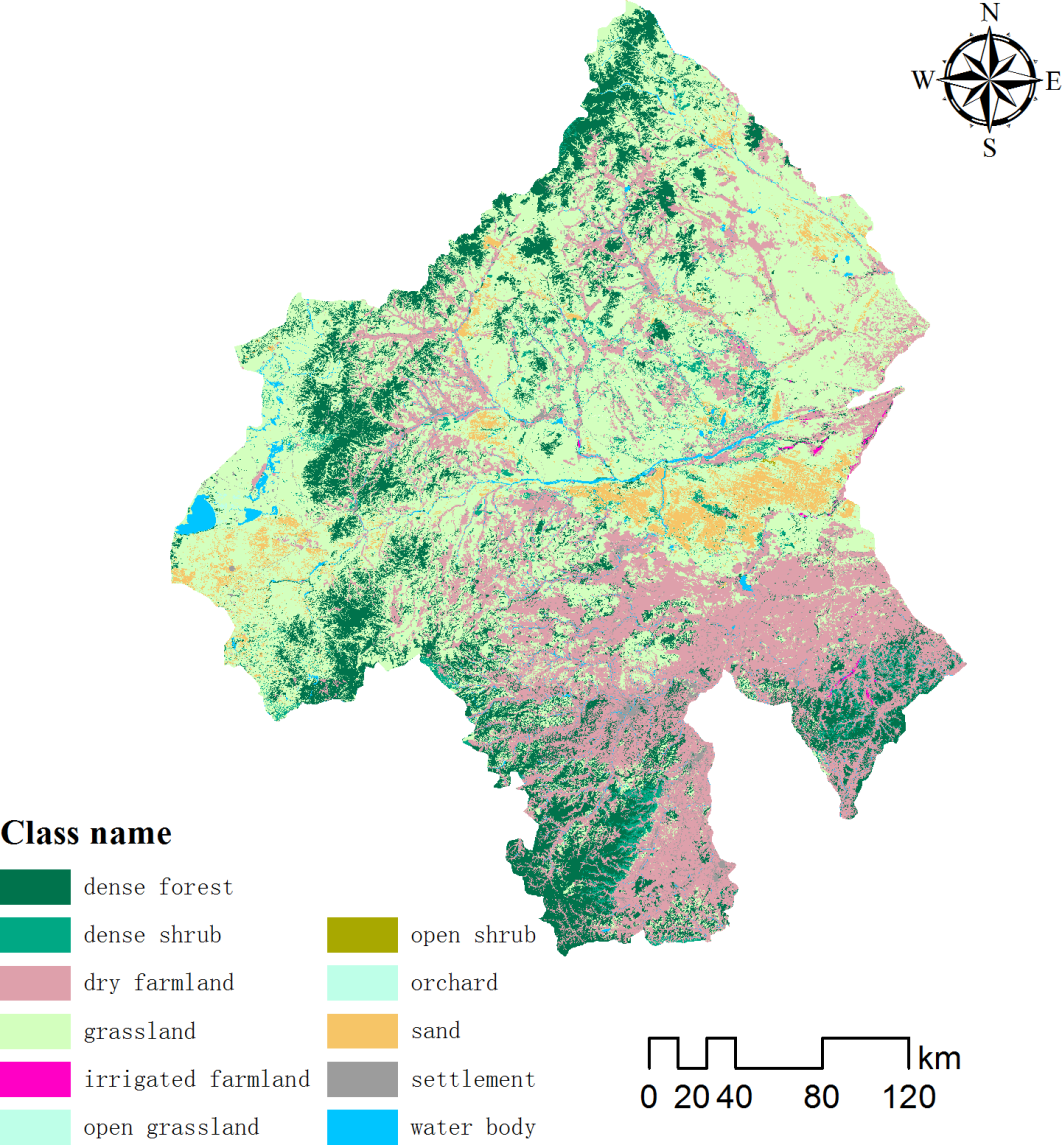
Map of land use for the study area.

The land use of a watershed influences runoff and is also an important indicator for the selection of suitable sites for harvesting rainwater. Land-use data were mainly derived from a second national land survey in China, at a scale of 1:1 000 000 [27]. Land use was divided into 11 categories based on the land-use classification of the US Bureau of Land Management (Fig 4). The majority of the area comprised grassland followed by forest (Table 2). Forest and grassland ecosystems were suitable sites for potential RWH in the study area.

**Table 2:** Land uses in the study area.

S no.	Land use	% Area	Main units	Suitability
1	Grassland	45.97	Grassland, meadows and grass clusters	Suitable
2	Forest	17.43	Deciduous broad-leaved and coniferous forests	Suitable
3	Dense shrubland	3.69	Primarily deciduous broad-leaved bushes	Suitable
4	Open shrubland	0.007	Primarily deciduous broad-leaved shrubs	Suitable
5	Open grassland	0.056	Weeds	Suitable
6	Orchard	0.005	Primarily arbor gardens	Suitable
7	Dry farmland	25.37	Corn, wheat, millet, beans	Unsuitable
8	Irrigated farmland	0.098	Rice	Unsuitable
9	Settlement	2.00	Residential, industrial and mining land	Unsuitable
10	Water body	1.48	Rivers and lakes, wetlands	Suitable
11	Sand	3.87	Desert and sand dunes	Unsuitable

#### 2.2.4 Hydrological soil groups

The SCS-CN method is very sensitive to the value of CN. CN represents the runoff potential based on hydrological soil class, land cover, growing season and antecedent conditions of soil moisture. CN is often within the range 30–100. A higher CN indicates that less rainwater is absorbed by the soil and thus a larger amount of surface runoff. The difference in times of collection of monthly precipitation data at the various meteorological stations was small, so antecedent moisture conditions II was used for the entire research area for a given storm. The CN values for the study area were based on the US Engineering Handbook and related publications (Table 3). High CN values were mainly distributed in HSG-D mountainous areas in the western part of the Chifeng area, intermediate CN values were mainly distributed in HSG-B grasslands and meadows in the eastern part of the area and the lowest CN values were mainly distributed in HSG-A sandy lands.

**Table 3:** CN as a function of land use and hydrological soil group.

Land use	CN-II			
	HSG-A	HSG-B	HSG-C	HSG-D
Forest	36	60	73	79
Shrubland	35	56	70	77
Grassland	49	69	79	84
Open forest	48	67	77	83
Open grassland	68	79	86	89
Garden	45	66	77	83
Paddy field	79	85	89	91
Dry farmland	66	76	82	85
Settlement	81	88	91	93
Sand	30	30	30	30

Data sources: USDA NRCS, National Engineering Handbook; USDA NRCS, Urban Hydrology for small watersheds; Arc CN-Runoff software Attached List of CN values.

### 2.3 SCS-CN model

The SCS-CN model, developed by the US Soil Conservation Service in 1972, has been widely used internationally for estimating runoff and managing water resources due to its versatility [28,29]. Several important properties of this method, such as soil characteristics, land use, terrain slopes and antecedent soil moisture conditions, are taken into consideration [30]. The SCS-CN method was originally designed for use in small watersheds, but it has been modified for application to larger areas by weighting CNs based on the landuse/landcover of the area under study [31]. The ArcCN-Runoff tool, an extension of ArcGIS, is a simple and accurate method to measure surface runoff. The SCS-CN model was designed for any shape of landuse/landcover and soil polygon, and the use of the ArcCN-Runoff tool facilitates better runoff prediction. A detailed, stepwise calculation of runoff by the ArcCN-Runoff tool in ArcGIS is described by Zhan and Huang [22]. The main operation is: 1) cut the boundary diagram into maps of soil and land-use types in the watershed, 2) use the criteria for USDA soil classification to re-classify various soil types as HSG-A-D and 3) assign a CN value for the soil/land-use cross map. The model calculates:

$$Q=\frac{{\left(P-\lambda S\right)}^{2}}{P+(1-\lambda )S}\;\left(P<\lambda S\right)$$ (1)

Q=0(P>λS) (2)

S=25400/CN−254 (3)

Where: *Q* is the runoff volume (mm), *P* is the total rainfall (mm), λS is the initial loss of rainwater (mm), λ is a regional parameter (0.1≤λ≤0.3) mainly representing geological and meteorological factors, *S* is the maximum possible retention (mm) and CN is the curve number from 0 to 100. The empirical value of the output of the SCS-CN model is based on the data for rainwater runoff from large gentle slopes (about 5°). The most accurate value of λ is 0.2 when simulating surface runoff on gentle slopes <10° [32,33].

### 2.4 Universal soil loss equation

The universal soil loss equation (USLE) predicts a long-term average annual rate of erosion on a field slope based on rainfall pattern, soil type, topography, land cover and management practice. USLE is the most popular tool for assessing the risk of water erosion due to its modest data demands and transparent model structure [34]. The equation can also predict soil erosion for many land-use types [35]:

A=R×K×LS×C×P (4)

where: *A* is the calculated soil loss per unit area, *R* is an erosivity factor for the rainwater runoff, *K* is a soil-erodibility factor, *L* is a slope-length factor, *S* is a slope-gradient factor, *C* is a vegetation-management factor and *P* is a support-practice factor. We calculated *R* by analyzing the 20 years of rainfall data collected from the nine rain-gauge stations(S1 Fig). *K* values for the various soil types in Chifeng have been estimated from the USLE nomo-graph [36, 37] and then a detailed table (Table 4). The parameters of the *LS* factor were derived using DEM data(S2 Fig) and integrated into the ArcGIS environment using:

LS=power(flowaccumulation×cellsize/22.13,0.4)×power(slope×0.01745)/0.09,1.4)×1.4

A C-factor map was prepared based on the map of land use/land cover. P in Eq 4 represents the effects of practices that will reduce the amount and rate of runoff and thus the amount of erosion (Table 5).

**Table 4:** K for the various soil types.

Textural class	Silty clay	Clay	Silty clayey loam	Clayey loam	Silty loam	Loam	Sandy clayey loam	Sandy loam	Loamy sand	Sand
**K** **(t/ha)**	0.58	0.49	0.72	0.67	0.85	0.67	0.45	0.29	0.09	0.04

**Table 5:** P for the various land uses.

	Forest	Shrubland	Grassland	Farmland	Water	Settlement	Sand
**P**	1	1	1	0.7	0	0.25	0.2

## 3 Results and discussion

### 3.1 Runoff potential

The water budget for a watershed is the balance between rainfall and water loss by evapotranspiration, groundwater recharge and runoff [38, 39]. Runoff can easily be collected and conveniently stored, so it is one of the most important parameters for identifying potential RWH sites. The calculated average annual runoffs for the land uses and soil classes are provided in Table 4. The yearly runoff average was about 246.50 mm with a standard deviation of 34.3 mm. The average annual runoff potential for forest, shrubland and grassland were 252.90, 230.92 and 264.44 mm, respectively. The maximum runoff and average annual runoff potential in the southern Chifeng area were 342.30 and 277.65 mm, respectively. The eastern and western parts of the Chifeng area are on the periphery of Horqin Sandy Land and Hunshandake Sandy Land, where the surface runoff potential was the lowest and the average annual runoff potential was 178.98 mm, with a standard deviation of 18.3 mm. The low runoff yield in these areas was mainly due to lower rainfall, a larger sandy area and a higher infiltration rate.

**Fig 5:**
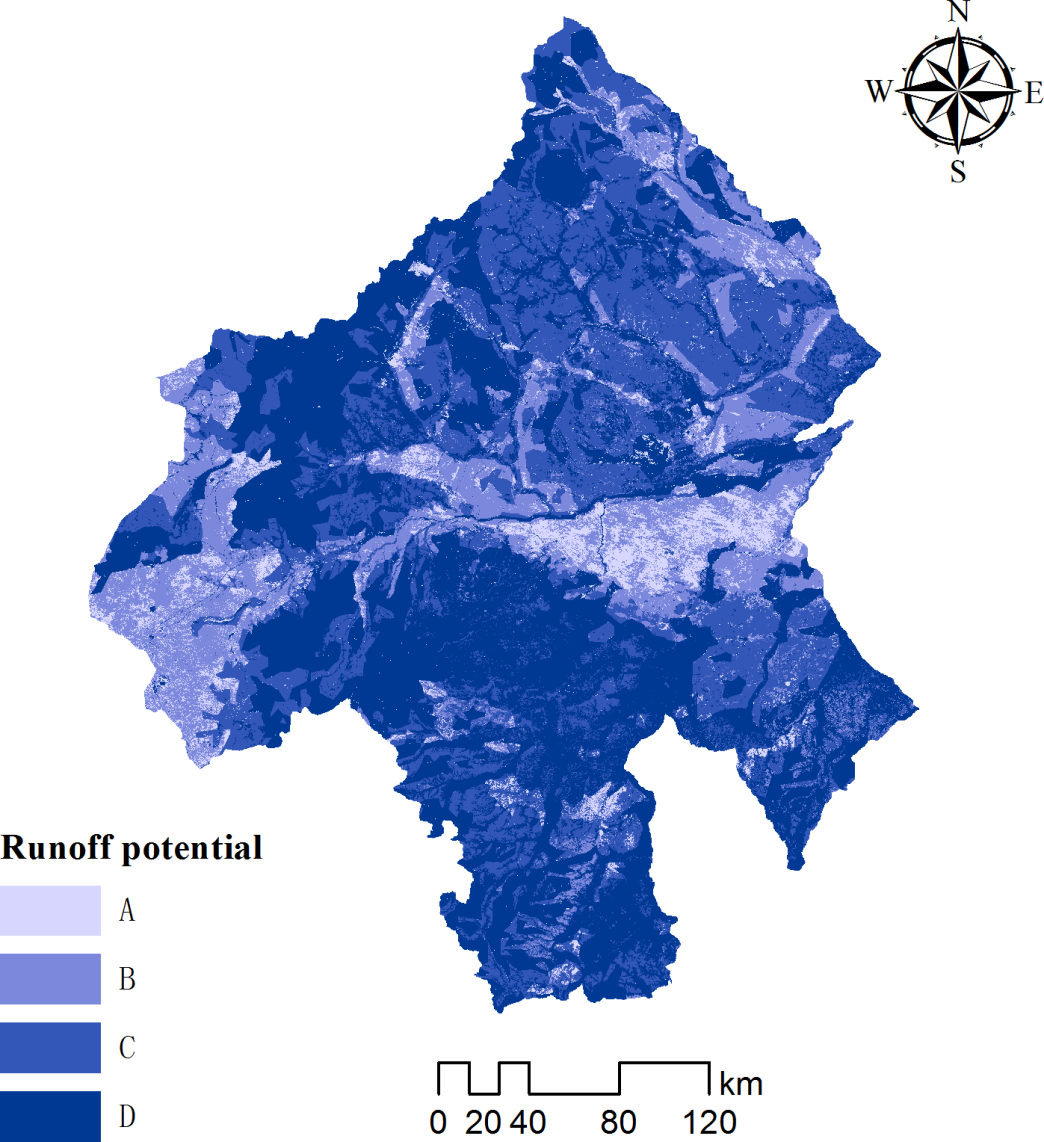
Map of runoff potential for the study area.

The average annual runoff was equally divided into Grades A-D from low to high, forming the runoff potential map (Fig 5). The zones with runoff potential Grades A-D accounted for 21.75, 17.53, 30.02 and 30.07% of the study area, respectively. High to moderate runoff potential zones were suitable for harvesting rainwater.

### 3.2 Soil and water erosion risk

**Fig 6:**
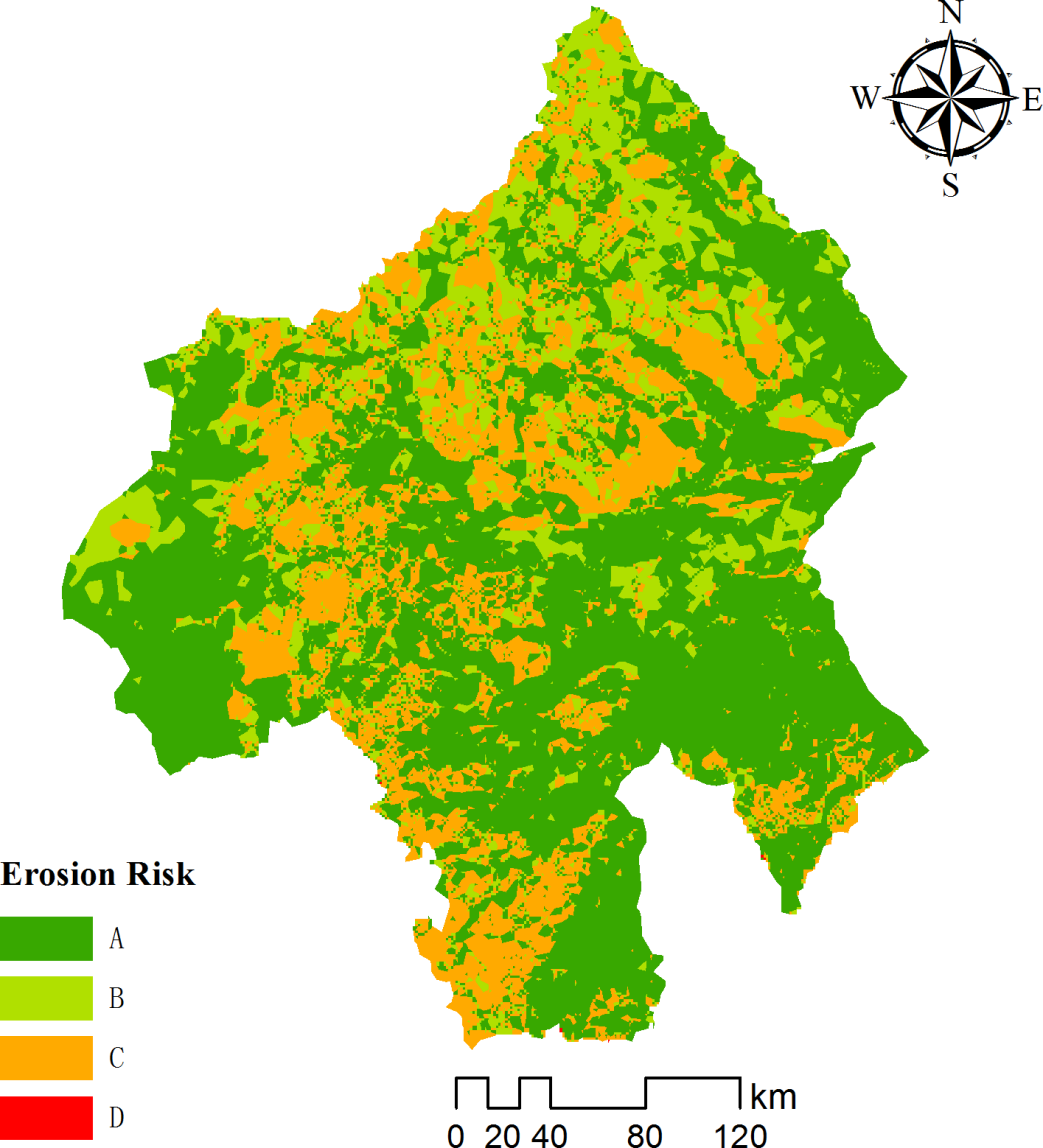
Map of the risk of soil and water erosion for the study area.

The transport of soil from one place to another can have several impacts, e.g. the openness of a land and the erosion of plant nutrients. The deposition of eroded soils can cause disturbances in waterways, e.g. deposition in rivers and reservoirs may decrease water quality. The average annual soil erosion (A) in the Chifeng area was assessed using USLE (Fig 6). The risk of soil erosion is closely associated with the meteorological environment, soil properties, topography and land use. Predicted average annual soil loss from the basin has been classified into four classes of erosion intensity (Table 5) to assess the potential severity of soil loss. About 57% of the area had a negligible to low risk of erosion, only 1% had a high risk of erosion and the rest of the area had a moderate risk of erosion.

Soil erosion is closely associated with rainfall characteristics, soil properties, topography and land use. Precipitation and surface runoff are the dominant factors affecting soil erosion. The greater the rain intensity and runoff, the greater the kinetic energy for eroding the soil and the higher the risk of erosion. Most of the soil is lost along streams and from steep slopes. The mountains and hills in the southern Greater Higgnan Mountains and northern Yanshan Mountains in the territory of Chifeng have an undulating terrain and contain a large amount of silty loam, so they have a relatively high risk of soil erosion; the plains in the eastern and western parts of the Chifeng area have a flat terrain, relatively low rainfall and thus a relatively low risk of soil erosion.

### 3.3 Sites suitable for harvesting rainwater

Harvesting rainwater involves the collection and management of rainwater runoff to increase water availability for domestic and agricultural use and for the maintenance of ecosystems [40]. To resolve the pressing problem of water shortage in the study area, we focused on increasing the collection of rainwater runoff, also taking into account soil and water conservation. The ecosystems of the study area dominated by moderate to high runoff and by moderate to low risk of soil erosion contained suitable RWH sites. The RWH sites were divided into Groups I-IV: Group I represents high surface runoff (Grade D) and low erosion risk (Rank A), Group II represents high surface runoff (Grade D) and relatively low erosion risk (Rank B), Group III represents moderately high surface runoff (Grade C) and low erosion risk (Rank A) and Group IV represents secondary high surface runoff (Grade C) and relatively low erosion risk (Rank B).

**Fig 7:**
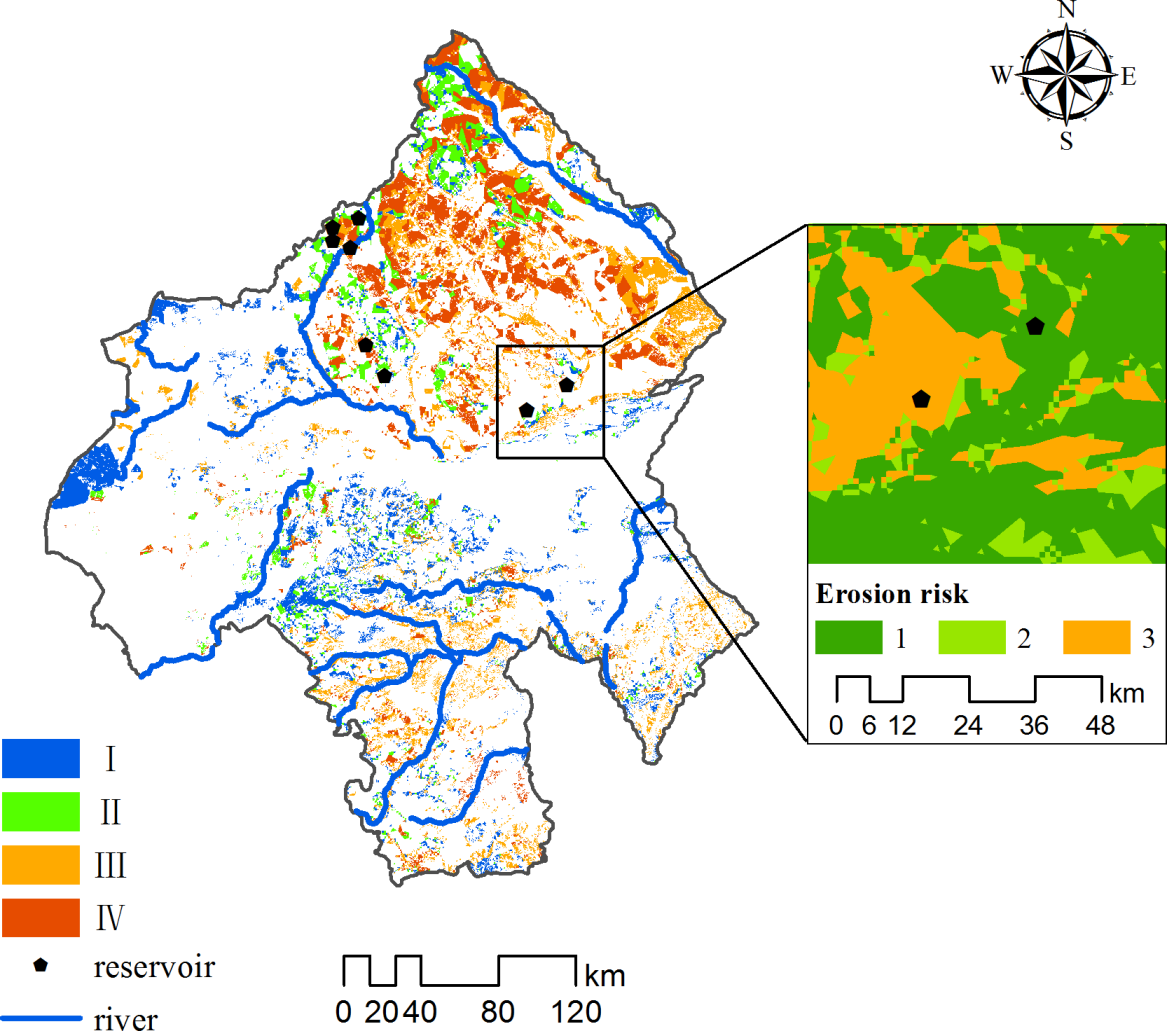
Map of potential sites for harvesting rainwater indicating analytically derived sites and sites for reservoirs validated by ground truth. (See S1 Table for reservoirs information).

The sites suitable for harvesting rainwater covered an area of 24.90%, whereas the remainder of the study area was determined unsuitable for harvesting rainwater. Optimal Group I and II RWH sites covered a small area, accounting for 8.4% of the study area, and sub-optimal Group III and IV RWH sites covered a larger area, accounting for 16.39% the study area. Grassland is the main land use in the study area, so a high proportion of the RWH area was grassland, and a lower proportion was shrubland (Table 6). The southern part of the Greater Higgnan Mountains in northern Chifeng had a large RWH area, the western and eastern parts of the Chifeng area belonging to Horqin Sandy Land and Hunshandake Sandy Land, respectively, had a smaller RWH area and the southern urban area had small and scattered RWH areas (Fig 7).

**Table 6:** Potential rainwater-harvesting areas.

Land use	Potential rainwater-harvesting sites (km^2^)			
	Group I	Group II	Group III	Group IV
Forest	285.25	125.99	742.11	256.18
Shrubland	75.8	54.66	38.57	9.05
Grassland	3060.31	2303.91	5750.58	5109.91
Total	4060.55	3553.4	8127.02	6635.24

Within the uncultivated ecosystem with areas of high runoff and low erosion risk, successful rainwater harvesting projects should account for allocation parameters [41,42], such as distance from rainfall collection and application areas, that influence the construction costs of RWH structures. Further research should determine the types of project suitable for the study area based on economic factors and target water supply. The specific engineering constraints should refer to Technical Code for Rainwater Collection, Storage and Utilization [43].

### 3.4 Ground-truth verification

Reservoirs are one of the most common techniques for harvesting rainwater. We selected the eight reservoirs on the Xilamulun River and its main tributary, the Chaganmulun River, as verification points. Accuracy was assessed by overlaying the existing structures identified in a ground-truth survey on the map of potential RWH. Seven of the reservoirs were within the simulated important RWH areas, four of which were in Group I and II RWH sites. Approximately 87.5% of the existing structures could be matched with the prediction map. Such a high accuracy of the study suggests good guidance for field implementation.

One of these reservoirs was outside the potential RWH sites, mainly because it was in an area with a high risk of soil erosion (Fig 7). Water and soil resources can be lost in large areas with serious soil erosion near water resources, which would deplete the soil, deposit sediments in reservoirs and severely contaminate water resources. Measures to conserve soil and water should therefore be applied upstream of this and similar reservoirs that have been completed to improve vegetation coverage and increase water storage and the capacity of ecosystems to conserve soil. Avoiding the establishment of reservoirs in large areas with a high risk of soil erosion, protecting water resources and guaranteeing reservoir water quality and quantity are important.

### 3.5 Future management of harvested rainwater

The relationship between potential RWH sites and other zones of ecological function were fully taken into account. Arid and semi-arid regions have limitations in potential runoff availability, and using ecosystems to harvest runoff is an important way to resolve the problem of regional water shortages [6, 10]. The blind pursuit of maximizing water resources, however, may cause soil erosion and thus soil and nutrient loss, so other ecological functions should be taken into account when harvesting rainwater resources. Gao Jixi (2015) suggested that a watershed with a complete ecological structure and coordinated functions should perform ecological functions such as floodwater storage, wastewater purification, product delivery and water, soil and biodiversity conservation [44]. Mapping Green Infrastructure of Pan-European (2015), China’s National Ecological Function Zoning (2015) and other plans have also divided zones of ecological function for water-flow regulation, water purification, erosion protection and other ecological functions. These plans require that available water resources be harvested by decreasing the consumption of water by vegetation and increasing surface runoff in areas with water shortages; surface runoff should be reduced by improving the soil, increasing vegetation coverage and biodiversity, establishing dams for storing silt and sediments and other measures of water and soil conservation in areas sensitive to soil erosion.

Erodibility is an important consideration, because sediment deposition can fill RWH structures, thus decreasing the volume of reservoirs and the quality of the water [45]. We linked problems of sediment accumulation with assessments of erosion potential, which is reasonable, but the spatial distribution of sediment deposition should be studied further to forecast problematic areas of sediment accumulation. The transport and deposition of eroded sediments from areas with high erosion potential to areas with low potential for RWH also constitute a major portion of eroded soil [46]. Models such as WaTEM-SEDEM, AnnAGNPS, ANSWERS and SWAT can estimate the spatial distribution of sediment deposition. Further research should delineate sediment deposition after soil has been eroded to improve the rationality and accuracy of the results.

The quality of harvested rainwater can be improved by measures of vegetation protection and management in potential RWH sites. Most areas of northern China suffer from severe drought, where vegetation evapotranspiration consumes more than 70% of the rainwater resources, especially in Inner Mongolia where our study was conducted; this proportion has exceeded 86% [47,48]. Forest density and vegetation type can determine the amount of rainwater intercepted and the volume of evapotranspiration, which in turn can affect effective precipitation and the amount of rainwater harvested. Cultivating vegetation with low water consumption, selecting appropriate vegetation densities and implementing scientifically based measures such as pruning and intermediate cutting are therefore important for improving RWH in areas where water resources are released.

Potential RWH should ensure the balance and equal distribution of water resources across a watershed or continuous watersheds. The change of any ecological unit in the watershed can be transferred to other areas by the ecological medium, i.e. water, representing the continuity of ecological processes and integration of ecological functions, and the balance and compatibility upstream and downstream of the watershed. RWH facilities can change the allocation of water resources. For example, Zhang Kai et al. (2008) found that the interception and excessive consumption of water resources midstream in the basin of the Heihe River, China’s second largest inland river, sharply decreased the amount of water in the lower reaches and led to a record low surface runoff [49]. The location, quantity and capacity of RWH facilities were thus further optimized based on the runoff available after storage by existing structures and also based on the carrying capacity of the basin’s water resources, but the study area could not be confined to small watersheds. RWH should be discussed from the perspective of large areas and watersheds to promote the efficient use and rational distribution of water resources.

## 4 Conclusions

Harvested rainwater is an alternative source of water in arid and semi-arid regions around the world. We selected the Chifeng area of Inner Mongolia in China as a representative semi-arid region with a monsoon climate. We developed and assessed a methodology that integrates a procedure of continuous runoff accounting based on the SCS-CN model with risk of soil erosion based on the USLE to identify sites suitable for harvesting rainwater. This area had moderate to high runoff potential and moderate to low risk of erosion, which is suitable for potential RWH.

Our results indicated that 24.90% of the study area was suitable for potential RWH. Optimal Group I and II RWH sites accounted for 8.4% of the study area. The verification results for reservoirs on the Xilamulun River and its main tributary, the Chaganmulun River, indicated that the accuracy of the method was 87.5% by comparing the existing structures with the map of potential RWH structures. The methodology was effectively demonstrated as a better technique, which had high accuracy and the ability to identify potential RWH sites in large geographical areas. This study has provided an important basis for establishing RWH facilities and alleviating water crises in arid and semi-arid areas.

## Supporting information

S1 Fig Average annual rainfall of Chifeng area. (ZIP)

S2 Fig Digital elevation model data of Chifeng area. (ZIP)

S1 Table Ground verficition information on reservoirs. (DOC)
