# Supplementary material for: Identifying important ecological areas for potential rainwater harvesting in the semi-arid area of Chifeng, China
Source: PLoS One. 2018 Aug 22;13(8):e0201132. doi: 10.1371/journal.pone.0201132 (PMC6104916; doi:10.1371/journal.pone.0201132)
Supplement: S1 Table — (DOC) [file pone.0201132.s003.doc]

| Reservoir No. | Reservoir name | Watershed | Latitude  (N) | Longitude (E) | Catchment  area( km^2^) | [Capacity](../../../../C:/Users/Administrator/AppData/Local/youdao/dict/Application/7.5.2.0/resultui/dict/%3Fkeyword=capacity)  ( m^3^) |
| --- | --- | --- | --- | --- | --- | --- |
| 1 | Hongwei | Chaganmulun | 44.35 | 118.53 | 46.6 | 1444800 |
| 2 | Wenggenshan | Chaganmulun | 43.53 | 118.75 | 7656 | 1239900 |
| 3 | Ashanhe | Chaganmulun | 44.25 | 118.63 | 162 | 430000 |
| 4 | Changxing | Chaganmulun | 44.36 | 118.68 | 44 | 500000 |
| 5 | Kedehe | Xilamulun | 44.39 | 118.45 | 17.2 | 181000 |
| 6 | Yongjunaimin | Xilamulun | 43.88 | 118.63 | 121 | 3584000 |
| 7 | Suji | Xilamulun | 43.48 | 119.78 | 913 | 8610000 |
| 8 | Baganuur | Xilamulun | 43.45 | 119.66 | 711 | 9430000 |

Supporting information

S1 Table. Ground verficition information on reservoirs.
